# Assessment of knowledge, attitude, and practice toward first aid among female school educators in Riyadh, Saudi Arabia: a cross-sectional study

**DOI:** 10.3389/fpubh.2024.1482181

**Published:** 2024-10-24

**Authors:** Doaa Abdelrahman, Nouran Abdullah Aleyeidi, Huda Turki Alqahtani, Falak Nasser Aldosari, Tmadher Ghanam Al Shammari, Norah Khalid Alrusaini, Rnda Fahad Almahfouz, Sarah Ali Altamimi, Shatha Abdulwahab Alshehri, Rahaf Muqbel Alsubaie, Raghad Abdulrahman Almuzaini, Jazil Abdulrahman Innab, Yara Abdulaziz Alsuhaibani

**Affiliations:** ^1^Department of Internal Medicine, College of Medicine, Princess Nourah Bint Abdulrahman University, Riyadh, Saudi Arabia; ^2^Department of Family and Community Medicine, College of Medicine, Princess Nourah Bint Abdulrahman University, Riyadh, Saudi Arabia; ^3^College of Medicine, Princess Nourah Bint Abdulrahman University, Riyadh, Saudi Arabia

**Keywords:** first aid, school, Saudi Arabia (KSA), knowledge, awarenes, school health (MeSH)

## Abstract

**Objectives:**

School-age children are constantly at risk of unintentional injuries. School educational staff are the primary group responsible for maintaining student welfare and responding to emergency situations. The present study aims to evaluate the knowledge and attitudes, practice level, and contextual factors related to first aid among female educational staff in Riyadh, Saudi Arabia.

**Methods:**

A cross-sectional study was conducted with a convenience sampling of 1,060 female school staff at all educational levels in Riyadh. Participants completed a self-administered online questionnaire that contained items assessing sociodemographic data, knowledge, attitudes, and practice regarding first aid.

**Results:**

Most of the 1,060 participants reported above average knowledge level, with a mean score of 21 (max 35). On the other hand, positive attitude toward first aid was high (*μ* = 22.9; max 25). Approximately 33.4% of staff had previous first aid training, and 79.2% were willing to participate in future trainings. First aid knowledge was highest for choking and lowest for seizures.

**Conclusion:**

The attitudes toward first aid were generally positive, and the knowledge of first aid was above average among most participants but still unsatisfactory. Mandatory first aid courses are necessary to support children’s health during their education.

## Introduction

First aid encompasses the initiated behaviors aimed at quickly resolving an acute illness or injury, relieving pain, and promoting recovery (1). Awareness toward first aid is essential to achieve solutions in case of accidents, but effective prevention and treatment of injuries first requires sufficient knowledge of first aid (2).

Accidental injuries can occur anywhere and at any time; however, in the case of children, many spend a substantial portion of their time at school. Some children may have health conditions such as epilepsy, asthma, and diabetes which are susceptible to worsening during their time at school (3). In addition, some emergency situations, such as hot weather and crowded environments, put children at risk of serious condition like heat stroke and syncope (4).

A study on French schoolchildren found that 52.8% of injuries occurred during physical activity, and 12.7% during school breaks (5). When a child experiences a sudden accidental injury during school, teachers and school instructors are the first to respond to the situation. Therefore, teachers and school instructors’ knowledge of first aid and its practice plays a significant role in preventing further injuries, complications from the injury, and even death (4).

In Saudi Arabia, approximately 41,561 fracture cases, 779 burn cases, 467 asphyxia cases, and 30,263 neck and back injuries were reported in schools in 2016 (6). A study conducted in 2019 assessing knowledge about first aid among male school teachers in Riyadh City found that only 14.9% of the study sample (436 participants) had good knowledge of first aid (defined as a score of 60% or higher) (7). Another study conducted in 2015 among Khamis Mushayt City secondary school teachers, which includes males and female teachers, found that only 19.6% of the 250 study participants were found to be knowledgeable about first aid (8). A more recent study conducted in 2021 in Hail City found 90.9% of male school teachers knew about first aid (6). However, the teachers’ attitude toward and practice of first aid have not been well reported in the aforementioned studies conducted in Saudi Arabia.

Few studies in Saudi Arabia have assessed school staff members’ knowledge of, attitudes toward, and practice of first aid, especially focusing on female staff members. This study aimed to investigate the level of knowledge, attitude toward, and practice of first aid among female school staff of all educational levels in Riyadh, Saudi Arabia. We also examined the factors affecting the level of knowledge of and attitude toward first aid among female school staff members.

## Methods

This was a cross-sectional online self-administered questionnaire-based study. A Research Electronic Data Capture (REDCap) tool – a secure web-based software platform designed to support data capture in research studies – was used to create the questionnaire and collect data from female school staff. This tool was hosted by Princess Nourah bint Abdulrahman University (PNU) (9). Questions regarding knowledge of, attitude toward, and practice of first aid were adopted and modified from previous studies (2–4, 6–8, 10–12). The questionnaire underwent face and content validity tests and was then tested in a pilot testing before use. The questionnaire included the following four sections:

1. Sociodemographic data, which included information on the staff’s age, marital status, level of education, years of working experience, type and level of school in which they were employed, and sources of first aid information.
2. Knowledge, which included seven sections designed to assess female school staff’s knowledge of first aid in seven different emergency situations: fainting, choking, epistaxis, seizure, difficulty in breathing, stabbing, and heat stroke. Each emergency situation had five questions with “true”/“false”/“I do not know” responses that assessed the staff’s knowledge of dealing with these situations. Each correct response was given one point for a total of 35 questions. Participants with scores that were 60% and above were considered knowledgeable.
3. Attitudes consisted of five questions that measured the school staff’s attitude toward first aid and their behaviors in such situations. Participants were asked to rate their agreement on a five-point Likert scale ranging from “strongly agree” to “strongly disagree.” For each question, a score of 5 was given for responses that showed strong agreement and a score of 1 was given for responses that indicated strong disagreement. The total number of points achievable was 25.
4. First aid practice consisted of 12 items measuring female educators’ practice of first aid. These items included statements that measured their readiness using yes, no, or neutral responses, such as, for example, maybe, I do not know, or not sure, depending on the question.

### Statistical analysis

All statistical analyses were conducted using SPSS software. The data management and analysis plan included descriptive statistics such as mean, standard deviation, frequencies, and percentages, which were used to describe the characteristics of the study sample. Quantitative data were analyzed using the t-test and one-way analysis of variance if normally distributed and using Mann–Whitney U test and Kruskal-Wallis test if not normally distributed, whereas the association of qualitative variables was analyzed using the chi-square test. Statistical significance was set at *p* < 0.05.

### Sample size

The sample size was calculated using Raosoft software using a 95% confidence level with an estimated 50% response distribution and a margin of error of ±5%. The minimum recommended sample size returned was 1,011. Assuming 5% of all participants will submit incomplete responses to the online survey, an additional 5% participants were added to the target samples size, yielding 1,062 as the target sample size.

### Sampling technique

Convenience sampling was performed by contacting those working in the education field through WhatsApp and encouraging them to pass it to others in the field until the required sample size was reached.

### Ethical considerations

Prior to data collection, approval was obtained from the Institutional Review Board of Princess Nourah Bint Abdulrahman University (PNU), Riyadh, Saudi Arabia (IRB log number: 22–1044). All the participants voluntarily participated in the study after receiving a clear description of the study objectives through an electronic informed consent form. All the participants had the right to withdraw from the study at any time.

## Results

We invited 1,670 educators to participate in the study, but we analysed only 1,060 responses after excluding those who declined to participate and incomplete responses. The relevant sociodemographic data is detailed in Table 1. Of the 1,060 participants, nearly half (582; 54.9%) were 40–49 years old, while 204 (19.2%) were 30–39 years old, 197 (18.6%) were 50 and above, and only 77 (7.3%) were 20–29 years old.

**Table 1:** Sociodemographic characteristics of study participants.

Characteristics	*n* (%)
Age	
20–29	77 (7.3%)
30–39	204 (19.2%)
40–49	582 (54.9%)
50-above	197 (18.6%)
Marital status	
Married	857 (80.8%)
Single/divorce/widowed	203 (19.2%)
Do you have children?	
Yes	881 (83.1%)
No	179 (16.9%)
Level of education	
High school/diploma	230 (21.7%)
Bachelor’s degree/master’s degree/Ph. D	830 (78.3%)
Role/job in school	
School agent/school manager	77 (7.3%)
Educational administrator	150 (14.2%)
Teacher	802 (75.7%)
Student supervisor/counselor	18 (1.7%)
School health instructor/school nurse	13 (1.2%)
Years of work Experience/service year	
10 years or less	349 (32.9%)
More than 10 years	711 (67.1%)
Type of school	
Government	894 (84.3%)
Private/International	166 (15.7)
Does the school employ a nurse?	
Yes	139 (13.1%)
No	921 (86.9%)
Source of information regarding first aid (more than one answer can be chosen)	
Books	222 (20.9%)
Internet/social media	765 (72.2%)
TV	274 (25.8%)
Training course	298 (28.1%)
Friends and relatives	259 (24.4%)
School level^*^ (more than one answer can be chosen)	
Kindergarten	215 (20.3%)
Primary	468 (44.2%)
Intermediate	305 (28.8%)
Secondary	308 (29.1%)

*Primary level is from grade 1 to 6, Intermediate is from 7 to 9 and secondary is from 10 to 12.

Most of the participants were married (857; 80.8%), had children (881; 83.1%), and had a bachelor’s degree or higher (830; 78.3%). Most participants were teachers (802; 75.7%), although educational administrators (150; 14.2%), school managers (77; 7.3%), student advisors (18; 1.7%), and school health instructors (13; 1.2%) also participated. At the school level, 29.1% of participants worked in high schools, 28.8% in secondary schools, 20.3% in kindergartens, and 44.2% worked in primary schools. Over half of the participants (711; 67.1%) had more than 10 years of experience in their role. Most of the participants (894; 84.3%) were from public schools and the vast majority (921; 86.9%) reported not having a nurse in their school.

Among all participants, 72.2% reported using the internet or social media to obtain information about first aid. Participants also reported getting first aid information from training courses (28.1%), through TV (25.8%), from friends and relatives (24.4%), and from books (20.9%).

**Figure 1:**
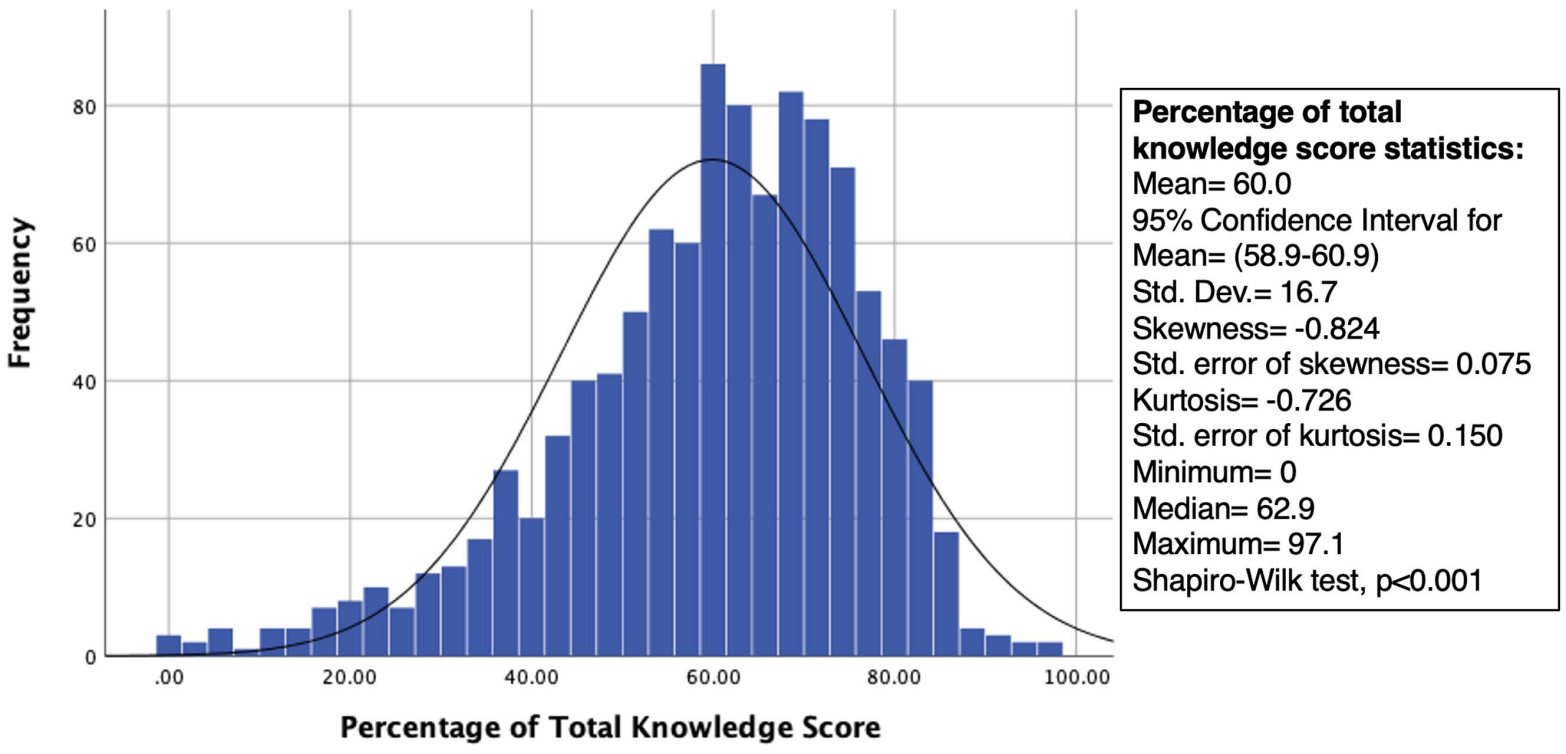
Frequency distribution of overall knowledge scores of female school educators in Saudi Arabia regarding first aid.

The mean knowledge score regarding first aid was 21 out of 35 which is equivalent to 60%, with an SD of 16.7. The minimum percentage correct was zero and the maximum was 97.1% (Figure 1). Participants who scored 60% correct and above (*n* = 632; 59.6%) were considered knowledgeable. This study assessed the participants’ knowledge of various medical emergencies, including fainting, choking, epistaxis, seizures, difficulty breathing, stabbing, and heatstroke (Table 2). Among the emergency situations assessed, the mean knowledge score for choking was the highest at 3.5 ± 1.2, indicating that participants had the most knowledge about choking compared to the other emergencies. Conversely, seizure had the lowest mean score at 2.6 ± 1.2, suggesting that participants had the least knowledge about seizures.

**Table 2:** The knowledge scores of the participants regarding first aid in different emergency situation.

Variables	Percentage of correct answers *n* (%)	Total mean score of correct answers out of five x̄ ± SD
If a student lost consciousness but still breathing, I should:		
Place the student on her side and bend her knee in a recovery position (True)	491 (46.3%)	3.1 ± 1.2
Slap her face or shake her to retrieve her (False)	769 (72.5%)	
Ask the rest of the students not to gather around her (True)	1,003 (94.6%)	
Spray perfume next to her face (False)	389 (36.7%)	
Make her drink water to wake her up (False)	655 (61.8%)	
If a student choked on something, I should:		
Call an ambulance immediately without intervention (False)	656 (61.9%)	3.5 ± 1.1
Give her multiple slaps on her back to make her eject the object (True)	515 (48.6%)	
Try to get the object out of her mouth with my hands (False)	743 (70.1%)	
Give the student multiple abdominal thrusts with your fist above her umbilicus (True)	998 (94.2%)	
Instruct the student to sit down and drink water to make her swallow the object (False)	883 (78.6%)	
If a student had epistaxis, I should:		
Leave the nose untouched until the bleeding stops (False)	924 (87.2%)	3.1 ± 1.4
Lie her flat and head facing upwards (False)	709 (66.9%)	
Turn her head downwards and press the lower soft area of the nose without blocking the airflow (True)	529 (49.9%)	
Turn her head downwards and press the top bony part of the nose (False)	463 (43.7%)	
Apply an ice pack on the nose (True)	674 (63.6%)	
If a student had a seizure, I should:		
Give her any drink or food until her awareness is restored (False)	880 (83.0%)	2.6 ± 1.2
Try to stop her movements during seizure to prevent her from harming herself (False)	320 (30.2%)	
Cushion the head if she is on the ground and loosen any tight clothing around the neck (True)	785 (74.1%)	
Apply a piece of clothing in her mouth to prevent her from biting her tongue (False)	69 (6.5%)	
Let her lie on her side and wipe away the excess drool after the spasms are over (True)	697 (65.8%)	
If a student was having difficulty breathing, I should:		
Use her asthma inhaler if she has it (True)	894 (84.3%)	2.9 ± 1.3
Encourage the student to breathe faster (False)	489 (46.1%)	
Lie the student on her back with a cushion under her head (False)	356 (33.9%)	
Let the student sit with a slight lean forward (True)	456 (43.0%)	
Instruct the student to breathe slowly by inhaling from her nose and exhaling from her mouth (True)	869 (82%)	
In case a student was stabbed with a sharp object, I should:		
Withdraw it immediately (False)	630 (59.4%)	3.0 ± 1.6
Bandage around the sharp object to prevent its movement (True)	450 (42.5%)	
Withdraw the sharp object when the bleeding stops (False)	548 (51.7%)	
Leave the object in place and seek further medical attention (True)	784 (74.0%)	
Press on the sharp object (False)	755 (71.2%)	
If a student had a heatstroke, I should:		
Move her to cooler place and remove excess clothing (True)	860 (81.2%)	2.7 ± 1.1
Applying ice pack or cold towel on her neck or underarm (True)	596 (56.2%)	
Give the student an antipyretic medication (False)	394 (37.2%)	
Give the student water to keep hydrated (False)	72 (6.8%)	
Seek urgent medical help (True)	949 (89.5%)	

As presented in Table 3, most participants showed a positive attitude toward learning first aid (about 89.5% of participants strongly agreed on the importance of learning the basics of first aid) and administering it (76% of participants strongly agreed with the idea that bystanders, and not just medical professionals, can perform first aid in case of emergencies). Approximately three quarters of the participants (76.1%) strongly agreed that if they had adequate first aid knowledge and skills, they would administer it to people in need. Around half (54.2%) of the participants strongly agreed and 21.4% agreed that providing first aid care to students in need was the responsibility of school staff. However, 4.2% strongly disagreed that school staff were responsible for providing first aid and 12.9% were neutral to the idea. Finally, 77.5% of the participants strongly agreed that learning first aid was important to them.

**Table 3:** Participants’ attitude toward first aid.

Prompt	Strongly disagree	Disagree	Neutral	Agree	Strongly agree
	*n* (%)	*n* (%)	*n* (%)	*n* (%)	*n* (%)
I think that learning the basic steps of first aid is important.	25 (2.4%)	4 (0.4%)	23 (2.2%)	48 (4.5%)	949 (89.5%)
Not only medical professionals but bystanders can also perform first aid during emergencies.	17 (1.6%)	16 (1.5%)	42 (4%)	159 (15.0%)	815 (76.9%)
First aid must be provided to people in need if you have the proper knowledge and skill.	20 (1.9%)	10 (0.9%)	33 (3.1%)	179 (16.9%)	807 (76.1%)
It is the responsibility of the school staff to administer first aid to students in need.	45 (4.2%)	65 (6.1%)	137 (12.9%)	227 (21.4%)	575 (54.2%)
It is important for school staff to learn first aid.	20 (1.9%)	13 (1.2%)	35 (3.3%)	159 (15%)	822 (77.5%)

Table 4 displays the level of first aid practice reported by the participants. Nearly two thirds (66.6%) had no prior first aid training. Among those that reported prior first aid training, 7.3% participated in a first aid course within the previous 2 years, and 26.1% participated in first aid training more than 2 years ago. Of those with prior first aid training, 79.9% were satisfied with the course, and 52.9% reported feeling confident to give first aid after the course. Also, about 79.2% (*n* = 814) of the participants were willing to take a training course in first aid, while 85.5% reported needing further education regarding first aid. This willingness to take first aid training was significantly higher among those who aged between 40 and 49, had lower level of education and working in governmental schools.

**Table 4:** Practice level of participants regarding first aid.

Variables	*n* (%)
When was you last first aid course training?	
Less than 2 years ago	75 (7.3%)
More than 2 years ago	269 (26.1%)
I have never trained before	685 (66.6%)
Are you satisfied with your first aid course training? (Only for those who received first aid course training *n* = 344)	
Yes	275 (79.9%)
No	69 (20.1%)
Are you confident in performing first aid after the course? (Only for those who received first aid course training *n* = 344)	
Yes	182 (52.9%)
No	162 (47.1%)
Are you willing to take first aid training course?	
Yes	814 (79.2%)
No	25 (2.4%)
Maybe	189 (18.4%)
Do you need further education regarding first aid?	
Yes	880 (85.5%)
No	25 (2.4%)
Maybe	124 (12.1%)
I have knowledge and experience of how to use each component in the first aid kit.	
Yes	192 (18.7%)
No	331 (32.2%)
Not sure	504 (49.1%)
Do you know where the school keeps the first aid kit?	
Yes	466 (45.4%)
No	424 (41.3%)
Not sure	136 (13.3%)
Does the first aid kit in your school cover the students’ needs?	
Yes	168 (16.4%)
No	332 (32.3%)
Maybe	527 (51.3%)
Have you ever encountered a situation where a student needed first aid?	
Yes	530 (51.5%)
No	279 (27.1%)
I do not remember	220 (21.4%)
Were you able to help a student with your information about first aid?	
Yes	560 (54.6%)
No	466 (45.4%)
How do you respond in emergency situations involving a student?	
I intervene according to what I deem correct in such a situation	929 (90.3%)
I do nothing	100 (9.7%)
What is the contact number in cases of health emergencies?	
999	125 (12.1%)
998	163 (15.8%)
997^*^	654 (63.6%)
993	87 (8.5%)

*997 is the correct number for health emergencies in Saudi Arabia.

Moreover, about half (49.1%) of the participants were not sure that they had the knowledge and experience of how to use each component in the first aid kit. However, 45.4% of the participants knew where the school stores the first aid kit, and nearly one sixth (16.4%) were certain that the first aid kit was sufficient.

In addition, 51.5% of the participants previously encountered an incident where a student at their school needed first aid, and 54.6% provided first aid to the student. Most of the participants (90.3%) did what they thought was correct when facing emergency situations involving a student, and more than half (63.6%) knew the correct number to contact in case of a health emergency.

Table 5 displays the knowledge and attitudes of the participants in relation to sociodemographic factors. Regarding the knowledge score, the highest level of knowledge of first aid was observed among health instructors/school nurses (23.9 ± 6.2; *p* = 0.005), in contrast to educational administers, who had the lowest scores (19.8 ± 5.9). Those with a bachelor’s degree, master’s degree, or PhD showed significantly higher levels of knowledge regarding first aid compared to those with a high school diploma or other diploma degrees (*p* = 0.047). The highest scores for knowledge was found in the group who had previously participated in a first aid training course (23.2 ± 5.4); the lowest scores were among those receiving information from friends and relatives (20.1 ± 5.8). Regarding school education level, the mean scores were approximately the same at each level. There were no statistically significant differences in the knowledge scores in terms of age, marital status, years of experience, type of school, or the presence of a nurse in the school in the study sample.

**Table 5:** Comparison between knowledge and attitude scores across sociodemographic characteristics.

Attached		Knowledge		Attitude	
		x̄ ± SD^*^	*p* value^**^	x̄ ± SD^*^	*p* value^**^
Age	20–29	20.9 ± 6.4	0.516	22.9 ± 3.3	0.013^***^
	30–39	21.2 ± 5.9		23.4 ± 3.1	
	40–49	20.7 ± 5.9		22.9 ± 3.3	
	50-above	21.4 ± 5.2		22.8 ± 3.9	
Marital state	Married	21.0 ± 5.6	0.224	23.0 ± 3.2	0.097
	Single/Divorced/Widowed	20.4 ± 6.4		22.4 ± 3.9	
Do you have children?	Yes	21.1 ± 5.7	0.119	23.1 ± 3.1	0.034^***^
	No	20.2 ± 6.6		22.3 ± 3.9	
Level of education	High school/Diploma	20.2 ± 6.1	0.047^***^	22.4 ± 3.8	0.010^***^
	Bachelor’s degree/master’s degree/PhD	21.1 ± 5.7		23.1 ± 3.2	
Role/Job at school?	School agent/school manager	22.0 ± 5.0	0.005^***^	22.9 ± 3.9	0.759
	Educational administrator	19.8 ± 5.9		22.8 ± 3.2	
	Teacher	20.9 ± 5.8		23.0 ± 3.3	
	Student supervisor/counselor	23.5 ± 3.8		22.0 ± 5.0	
	School health instructor/school nurse	23.9 ± 6.2		23.0 ± 2.2	
Years of experience	10 years or less	21.1 ± 5.9	0.249	22.9 ± 3.4	0.349
	More than 10 years	20.8 ± 5.7		22.9 ± 3.3	
Type of school	Government	20.9 ± 5.7	0.340	22.9 ± 3.4	0.199
	Private/International	21.1 ± 6.3		23.1 ± 3.1	
Does the school employ a nurse?	Yes	21.6 ± 5.7	0.110	23.3 ± 2.9	0.218
	No	20.8 ± 5.8		22.9 ± 3.4	

^*^The *p* value was calculated using an Mann–Whitney U test or Kruskal-Wallis test according to the number of categories. ^**^x̄= mean; SD = standard deviation. ^***^p < 0.05 was considered significant.

The highest knowledge score achievable was 35 and the highest attitude score achievable was 25.

Regarding the scores for attitude toward first aid, there was a significant difference between age groups (*p* = 0.013), with the highest scores among those aged between 30 and 39 years. Participants were not significantly different depending on their marital status (*p* = 0.097). However, educational staff who were married had slightly higher attitude scores compared to their non-married counterparts. Having children was found to have a positive association with their attitude. This difference was statistically significant (*p* = 0.034). Those with bachelor’s degrees, master’s degrees, and PhD had more favorable attitudes toward first aid than those with high school diplomas and other diploma degrees (*p* = 0.010). School education level did not appear to be associated with the source of information regarding first aid. There were no statistically significant differences in the attitude score for this attribute according to role in school, years of experience, type of school, or whether the school had a nurse.

Table 6 displays the relationship between participants’ knowledge and practice level. There was a statistically significant difference between knowledge levels in participants who had received training less than 2 years prior (56; 74.7%) and those who had received it more than 2 years ago (190; 70.6%). Surprisingly, among those who had not received any training before, 54% could be considered knowledgeable. Also, the participants who were satisfied with their first aid course, had statistically significant better knowledge about first aid (205; 74.5%). Interestingly, we found that the participants who had more knowledge self-reported more willingness to take more first aid training (528; 64.9%) and reported needing more education regarding first aid (547; 62.2%). Most knowledgeable participants had faced a situation with a student who needed first aid and were able to appropriately manage the situation.

**Table 6:** Relationship between participants’ first aid knowledge and practice.

Variable		Knowledgeable Participant^*^ *n* = 632	Not knowledgeable participant *n* = 424	*p* value^**^
		*n* (%)	*n* (%)	
When was your last first aid course training?	Less than 2 years ago	56 (74.7%)	19 (25.3%)	<0.001^***^
	More than 2 years ago	190 (70.6%)	79 (29.4%)	
	I have never trained before	370 (54.1%)	314 (45.9%)	
Are you satisfied with your first aid course training? (Only for those who received first aid course training)	Yes	205 (74.5%)	70 (25.5%)	<0.013^***^
	No	41 (59.4%)	28 (40.6%)	
Are you confident in performing first aid after the course? (Only for those who received first aid course training)	Yes	146 (80.2%)	36 (19.8%)	<0.001^***^
	No	100 (61.7%)	62 (38.3%)	
Are you willing to take first aid training course?	Yes	528 (64.9%)	286 (35.1%)	<0.001^***^
	No	9 (36.0%)	16 (64.0%)	
	Maybe	78 (41.5%)	110 (58.5%)	
Do you need further education regarding first aid?	Yes	547 (62.2%)	333 (37.8%)	0.001^***^
	No	10 (40.0%)	15 (60.0%)	
	Maybe	59 (48.0%)	64 (52.0%)	
I have knowledge and experience of how to use each component in the first aid kit.	Yes	140 (72.9%)	52 (27.1%)	<0.001^***^
	No	188 (57.0%)	142 (43.0%)	
	Not sure	287 (56.9%)	217 (43.1%)	
Do you know where the school keeps the first aid kit?	Yes	304 (65.2%)	162 (34.8%)	0.001^***^
	No	237 (56.0%)	186 (44.0%)	
	Not sure	73 (53.7%)	63 (46.3%)	
Does the first aid kit in your school cover the students’ needs?	Yes	101 (60.1%)	67 (39.9%)	0.035^***^
	No	217 (65.4%)	115 (34.6%)	
	Maybe	297 (56.5%)	229 (43.5%)	
Have you ever encountered a situation where a student needed first aid?	Yes	342 (64.7%)	187 (35.3%)	<0.001^***^
	No	174 (62.4%)	105 (37.6%)	
	I do not remember	100 (45.5%)	120 (54.5%)	
Were you able to help a student with your information about first aid?	Yes	375 (67.0%)	185 (33.0%)	<0.001^***^
	No	240 (51.6%)	225 (48.4%)	
How do you respond in emergency situations involving a student?	I intervene according to what I deem correct in such a situation	577 (62.1%)	352 (37.9%)	<0.001^***^
	I do nothing	39 (39.4%)	60 (60.6%)	
What is the contact number in cases of health emergencies?	999	68 (54.4%)	57 (45.6%)	0.274
	998	97 (59.5%)	66 (40.5%)	
	997^**#**^	404 (61.9%)	249 (38.1%)	
	993	47 (54.0%)	40 (46.0%)	

^*^Participants with scores that were 60% and above were considered knowledgeable. ^**^p value was calculated using the chi-square test. ^***^p < 0.05 was considered significant. ^**#**^997 is the correct number for health emergencies in Saudi Arabia.

## Discussion

First-aid awareness among school educational staff is crucial to help save children who face many injuries. This study assessed participants’ knowledge regarding first aid in various medical emergencies such as fainting, choking, epistaxis, seizures, difficulty in breathing, stabbing, and heat stroke. The overall mean knowledge score was above average, with an average score of 21.0 (*n* = 1,056). These findings were inconsistent with AlYahya et al. in 2019 (*n* = 436), whose study on male teachers showed a mean score below the average 10.63 out of 25 (41.4%). In the same study, those who scored 60% and above correct were only 14.9% of the study sample, while in the current study 59.6% of participants did so (7). This might be due to differences in the sample and in the knowledge assessment questions.

Notably, the highest score for knowledge of first aid was for responding to choking events, and the lowest mean score for knowledge was for responding to seizures. This might be explained by more frequent personal experiences with choking cases, considering its higher frequency among school-aged children. In contrast to the current study, a study in Jeddah, Saudi Arabia aimed to evaluate the knowledge of first aid in response to epilepsy and seizure among teachers at all educational levels, and found that most participants correctly answered questions assessing their responses during and after a seizure attack (10).

Despite their average knowledge, most participants showed a positive attitude toward learning and providing first aid. About 89.5% of participants strongly agreed on the importance of learning the basics of first aid; these results were in line with those of another study conducted on male schoolteachers (7). Moreover, the current study also showed that roughly two-thirds of the participants did not receive any first-aid training. This result was in line with AlYahya et al. (7) who showed that more than two-thirds of the male teachers did not take any previous first-aid training, indicating a shortage of first-aid courses in this region for both genders due to the lack of first-aid courses and not making them mandatory for the academic staff. The findings also revealed that the knowledge of participants who received recent training in the previous 2 years was significantly better than that of those who received training more than 2 years ago. Similarly, a study in South India showed an inverse relationship between knowledge of first aid and the number of years since receiving first aid training (11).

Moreover, when participants were asked about encountering situations requiring first aid intervention, more than one half reported facing such situations and the proportion of school staff who managed to help with their first aid knowledge was more than 50%. This suggests that most participants felt their first aid training allowed them to intervene positively when they encountered a situation that required first aid at their school. This is counter to what is observed elsewhere in the literature as a study conducted in Ethiopia found that the majority of kindergarten teachers were exposed to situations that required first-aid, but very few performed the required activity (2).

In a study in Riyadh, Saudi Arabia showed that 71.6% of the male teachers knew the phone number of the local Red Crescent, and these findings are in agreement with our findings in females (63.6%) (7).

Surprisingly, years of experience were found to have an inverse relationship with participants’ knowledge, as those with 10 or fewer years of experience had slightly better knowledge than participants with more than 10 years of experience. Although not significant, these unexpected results might be explained by the differences in the participants’ age, as those with more than 10 years’ experience are older and might have less motivation to take first aid training. These findings contrast with those of previous studies, which revealed that those with more years of experience showed better knowledge levels than those with less years of experience (2, 7).

In our findings regarding the type of school in which the participants worked, the private, international and governmental school educators had no significant difference in their knowledge. This finding is not in agreement with the results of previous studies where the knowledge was better in private and international schools (2, 12).

In the current study, most of the participants’ main sources of information were the internet and social media. However, those who obtained information regarding first aid from formal course training had greater knowledge than those who received it from other sources. These findings were partially supported by Ganfure et al. (2), who found that teachers who gained information about first aid from health professionals and health institutes were twice as knowledgeable as those who received it from other sources.

The current study also showed that having a positive attitude toward first aid was not affected by participants’ years of experience with similar attitude scores for those with more than 10 years of experience compared to those with 10 years or fewer. This was in contrast to the Ganfure et al. (2) study, where the positive attitudes toward first aid of kindergarten teachers who had 5–10 years and more than 10 years of experience were five and four times higher, respectively, than that of teachers who worked for less than 5 years. These variations could be due to differences in school setups, sociodemographic characteristics of both samples, or differences in measurements.

Our study also demonstrated that participants with higher level of education showed higher score in knowledge and a more favorable attitude toward first aid. Also, those working as students’ supervisor/counselor, school health instructor or school nurse had the highest knowledge scores. In AlYahya et al. (7), they reported that the knowledge of first aid was not significantly better among teachers who had a higher educational level.

## Conclusion

This study indicated an acceptable level of first aid knowledge among most school staff. School staff’s knowledge was affected by their job in the school, their level of education and if they previously faced a situation that needed first aid use. We found that there was a positive attitude among school staff regarding first aid, especially among those who had children, had higher level of education or faced a situation that needed first aid use. There is a need for further and continuous training because nearly two-thirds of the participants had not been trained before in first aid, and the knowledge scores for first aid were higher among those who took first aid training less than 2 years ago.

### Limitations

This study has several limitations. First, as the survey was distributed online though a convenient sampling technique, it is possible that the sample does not represent the entire population of the school staff. Our study was conducted only with female participants, which may limit the generalizability of the study to male educators.

Another limitation of our study is that it was a questionnaire-based study; therefore, we could not evaluate the actual practice level of the participants. Further studies are required to address this issue. The lack of sufficiently similar studies conducted in the same region limits comparisons with other studies. However, this study will be helpful for the Ministry of Health and Education when planning first-aid courses for school staff.

### Recommendations

Based on our results, we recommend a first aid training course for educators every 2 years. The content of the course should cover all the aspects of first aid mentioned in this study which includes fainting, choking, epistaxis, seizure, difficulty in breathing, stabbing, and heat stroke. The current study’s knowledge questions can be used as a pre-post questionnaire or a written assessment method for the trainees after finishing the course in addition to a practical assessment method to evaluate their skills.

We also recommend further investigation to evaluate the practice level of first aid by conducting actual first aid skills assessment in school educational staff. In addition, we recommend the evaluation of the impact of different types of first aid training through intervention studies. Moreover, we suggest coordination between the Ministry of Health and Ministry of Education to train school staff and improve their skills in first aid response and emergency cases. Furthermore, we propose attracting qualified teachers to work as health educators in schools, setting up first-aid training programs, and providing staff with certificates of complement and rewards for their excellence.

## Data Availability

The raw data supporting the conclusions of this article will be made available by the authors, without undue reservation.

## References

[1] Zideman DA De Buck EDJ Singletary EM Cassan P Chalkias AF Evans TR. European resuscitation council guidelines for resuscitation 2015 section 9. First Aid Resusc. (2015) 95:278–87. doi: 10.1016/j.resuscitation.2015.07.031 26477417

[2] Ganfure G Ameya G Tamirat A Lencha B Bikila D. First aid knowledge, attitude, practice, and associated factors among kindergarten teachers of Lideta sub-city Addis Ababa, Ethiopia. PLOS ONE. (2018) 13:e0194263. doi: 10.1371/journal.pone.0194263, PMID: 29534091 PMC5849320

[3] Sharma R Kumar A Masih S. Knowledge and practice of primary school teachers about first aid management of selected minor injuries among children. Int J Med Public Health. (2014) 4:458. doi: 10.4103/2230-8598.144114

[4] Bakalarski P Chavada P Mazet G Combes D Dekesel B. Assessment of teachers’ knowledge about first aid. Crit Care Innov. (2020) 3:18–23. doi: 10.32114/CCI.2020.3.1.18.23

[5] Sapien RE Allen A. Emergency preparation in schools: a snapshot of a rural state. Pediatr Emerg Care. (2001) 17:329–33. doi: 10.1097/00006565-200110000-00003, PMID: 11673708

[6] Alshammari KO. Assessment of knowledge, attitude, and practice about first aid among male school teachers in hail city. J Family Med Prim Care. (2021) 10:138–42. doi: 10.4103/jfmpc.jfmpc_1322_20, PMID: 34017716 PMC8132825

[7] AlYahya IA Almohsen HA AlSaleem IA Al-Hamid MM Arafah AM Al Turki YA. Assessment of knowledge, attitude, and practice about first aid among male school teachers and administrators in Riyadh, Saudi Arabia. J Family Med Prim Care. (2019) 8:684–8. doi: 10.4103/jfmpc.jfmpc_316_18, PMID: 30984695 PMC6436304

[8] Al Gharsan M Alarfaj I. Knowledge and practice of secondary school teachers about first aid. J Family Med Prim Care. (2019) 8:1587–93. doi: 10.4103/jfmpc.jfmpc_76_19, PMID: 31198719 PMC6559104

[9] Harris PA Taylor R Minor BL Elliott V Fernandez M O’Neal L. The REDCap consortium: building an international community of software platform partners. J Biomed Inform. (2019) 95:103208. doi: 10.1016/j.jbi.2019.103208, PMID: 31078660 PMC7254481

[10] Kanjo M Najjar A Bokhari AY Alqarni GA Darwesh EA Alqarni GS. Knowledge of epilepsy and seizure first aid among teachers in Jeddah, Saudi Arabia Epilepsy. Behav Rep. (2021) 16:100475. doi: 10.1016/j.ebr.2021.100475, PMID: 34505052 PMC8411200

[11] Joseph N Narayanan T Bin Zakaria S Nair AV Belayutham L Subramanian AM. Awareness, attitudes and practices of first aid among school teachers in Mangalore, South India. J Prim Health Care. (2015) 7:274–81. doi: 10.1071/hc15274, PMID: 26668832

[12] Workneh BS Mekonen EG Ali MS. Determinants of knowledge, attitude, and practice towards first aid among kindergarten and elementary school teachers in Gondar city, Northwest Ethiopia. BMC Emerg Med. (2021) 21:73. doi: 10.1186/s12873-021-00468-6, PMID: 34154534 PMC8215869

